# North-Eastern Hospital for Children

**Published:** 1902-05-17

**Authors:** 


### NORTH-EASTERN HOSPITAL FOR CHILDREN.

### MEMORIAL STONE LAID.

The trustees of the North-Eastern Hospital for Children, Hackney Road, have made a beginning towards the urgently needed new building; the memorial-stone was laid by H.R.H. Princess Henry of Battenberg on Thursday, the 8th inst., amid much enthusiasm, and the ceremony was witnessed by a large concourse of people. For a long distance along Hackney Road flags and bunting floated in the bitter north wind, and although rain fell every now and then, the street was lined on each side with people of Hackney, Bethnal Green, and Shoreditch, who cheered the Princess as she entered the marquee, and waited to see her again as she left the hospital. There was music to while away the time of waiting. Outside, the band of the Honourable Artillery Company; inside, the string band of the Scots Guards, and a quintette arranged by Mr. N. Vert.

The Princess, who was accompanied by her daughter, Princess Ina, was attended by a guard of honour of the Honourable Artillery Company under the command of Captain Hodges, and was received at the entrance by the Lord Bishop of London, Lord Frederick Fitz-Roy (chairman) and Lady Frederick Fitz-Roy, Lord William Cecil (chairman-elect of the general committee) and Lady William Cecil, Miss Phillips (foundress of the hospital), Mrs. Bischoffsheim (president of the building fund committee), and other ladies and gentlemen. Bouquets were presented to the Princesses by a little patient, the National Anthem was played, and the mayors and mayoresses representing the three boroughs of Bethnal Green, Hackney, and Shoreditch were presented. Prayers were read by the Bishop, the hymn "Thou to whom the sick and dying" was sung, and then Lord Frederick Fitz-Roy thanked the Princess, on behalf of the governors, for her presence, and asked Mr. Charles G. J. Port, chairman of the building committee, to read the address.

#### Historical.

The hospital, said Mr. Port, was founded by two Quaker ladies in 1867, when the premises consisted of a small house in Virginia Road, Bethnal Green. In the following year a move was made to 127 Hackney Road, where the work was carried on for three years. The hospital at length found a more permanent home in two houses, Nos. 327 and 329, which until recently stood on the spot on which the ceremony was taking place. "The freehold of these two houses, and of others adjoining, having been purchased," the speaker continued, "the first building fund of the hospital was started, and the premises in Goldsmith's Row, now in use, were built at a cost of £18,000 between 1875 and 1880, being opened in the latter year by their Royal Highnesses the Duke and Duchess of Connaught. Since then no additional premises have been provided. Owing to the continually increasing number of patients and the greater space now required through the advance of medical and surgical science during the past twenty years, the need for largely increased accommodation has become extremely acute. The committee, after many efforts to obtain funds and after much careful consideration, have at length adopted a comprehensive scheme of enlargement, of which this new building forms the chief portion. The freehold of the further land required having been purchased at a cost of £7,600, the committee have in hand and in prospect about £17,000 for building purposes. The cost of the building which your Royal Highness is now inaugurating will be about £30,000, including furniture and fittings, and the sum of about £13,000 is therefore required to enable it to be opened next year free from debt."

### Advantages.

The new building, the speaker continued, would contain wards for 50 beds, a new operation room, accident room, bacteriological rooms, a nurses' dining-room, linen-rooms, and secretary's offices, etc. Plans were being prepared for a new out-patients' department, a separate isolation block, and a nurses' home. These additional buildings were roughly estimated to cost £20,000, so that the expenditure in new premises would come to about £50,000.

### Local Support.

It was gratifying to be able to state that the inhabitants of Bethnal Green, Shoreditch, Hackney, and the neighbouring districts took a keen interest in the welfare of the hospital, and provided about £1,000 in small contributions towards its maintenance. The visit of her Royal Highness, the speaker concluded, while greatly encouraging this valuable local support, would draw attention generally to the needs and claims of the hospital, and would greatly facilitate the accomplishment of the difficult task undertaken by the committee.

The architect, Mr. William C. Marshall, and the builder, Mr. Walter Nightingale, having been presented, the Princess laid the stone with the customary formalities.

When the bishop had pronounced the benediction, a number of children, chiefly little girls, went up on the platform and presented purses in aid of the building fund. Among others was a boy representing the boys of Columbia Road Board School, who had collected £10 10s.; and Charlie Brown, a patient, who gave a cheque for £250 on behalf of the *Gentlewoman* scheme, by which the children of the rich work for the children of the poor: this was to complete the endowment of a cot. The cot was afterwards named by the Princess as a memorial to Prince Christian Victor.

### Note.

The present hospital contains 57 beds, an out-patient hall (accommodation for 200 mothers with children), and two consulting-rooms and dressing-rooms. The population of the districts more especially served by the hospital is over 500,000. The new building was begun in November 1901, and is expected to be complete in the middle of January 1903. The building fund was started with a legacy of £5,000, received in 1893 under the will of the late Mr. John Horniman, who also left £5,000 to form the nucleus of an endowment fund. The amount since raised is about £20,000. The cost of maintenance, now £6,000 yearly, will be about £9,000 when the new building is completed. King Edward's Fund has repeatedly urged the committee to proceed with the building scheme; the grants to the building fund have amounted to £1,250. Roentgen rays, light treatment for lupus, and a special dental department are noteworthy features of this hospital for children.

